# Supplementary material for: Earthquake transformer—an attentive deep-learning model for simultaneous earthquake detection and phase picking
Source: Nat Commun. 2020 Aug 7;11:3952. doi: 10.1038/s41467-020-17591-w (PMC7415159; doi:10.1038/s41467-020-17591-w)
Supplement: Supplementary file 1 — Supplementary Information [file 41467_2020_17591_MOESM1_ESM.pdf]

Supplementary Information for:

Earthquake Transformer: An Attentive Deep-learning Model for Simultaneous Earthquake Detection and Phase Picking

Mousavi et al.

This PDF file contains:

- Supplementary Note 1
- Supplementary Table 1, 2
- Supplementary Figures 1-17

Supplementary Note 1
Matrices used in our study to measure the performance are:

$$Precision = \frac{tp}{tp+fp},$$

$$Recall = \frac{tp}{tp+fn},$$

$$Fscore = 2.\frac{Precision.Recall}{Precision+Recall},$$

where tp, fp, and fn are true positives, false positives, and false negatives respectively.

$$Mean\ Absolute\ Error = \frac{\sum_{i=1}^{n} |A_i - F_i|}{n},$$

$$Mean\ Absolute\ Percentage\ Error = \frac{1}{n}\sum_{i=1}^{n} |\frac{A_i - F_i}{A_i}|$$

where A_i, F_i, and n are true value, predicted value, and number of samples in the test set respectively.

Supplementary Tables

Supplementary Table 1: Parameters used to perform classical pickers on the test data. t_ma is the time in seconds of the moving average window for dynamic threshold. n_sigma is the standard deviation controls the level of threshold to trigger potential picks, t_win is the time in seconds of moving window to calculate kurtosis or CFn.

method	method	nsigma	t_win
AIC	3	8	-
FilterPicker	2	6	1
Kurtosis	4	5	1

Supplementary Table 2: Threshold values used for running each model on the test set. These have been selected best on the best F-score result and varies based on the model characteristics.

Algorithm	Detection Threshold	P Threshold	S Threshold
EqTransformer	0.5	0.3	0.3
PhaseNet	-	0.3	0.3
CRED	0.5	-	-
Yews	0.5	0.5	0.5
GPD	-	0.95	0.95
PickNet	-	0.7	0.5
PpkNet	-	0.2	0.2
DetNet	0.1	-	-
STA/LTA	1.25	-	-

Supplementary Figures

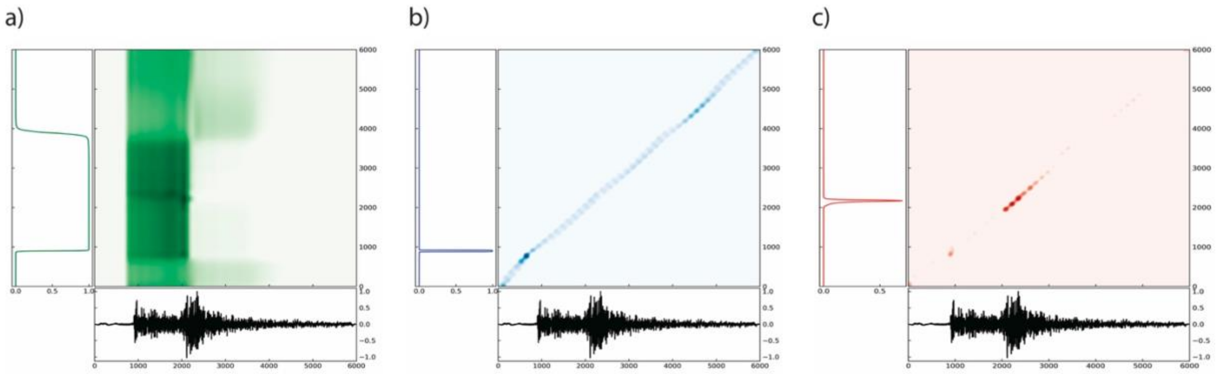

Supplementary Figure 1. The calculated energies (or scoring) for attention layers. This provides a measure of alignment, or match, between encoder and decoder states and is used by the decoder to decide focus. High energies indicate alignments of predicted probabilities and corresponding parts of waveform. Input waveform (bottom boxes), output prediction probabilities (left boxes), and corresponding scoring (central boxes) for a) transformer (I in Fig 1), b) local attention for P-phase (II in Fig 1), and c) the local attention for S-phase (III in Fig 1).

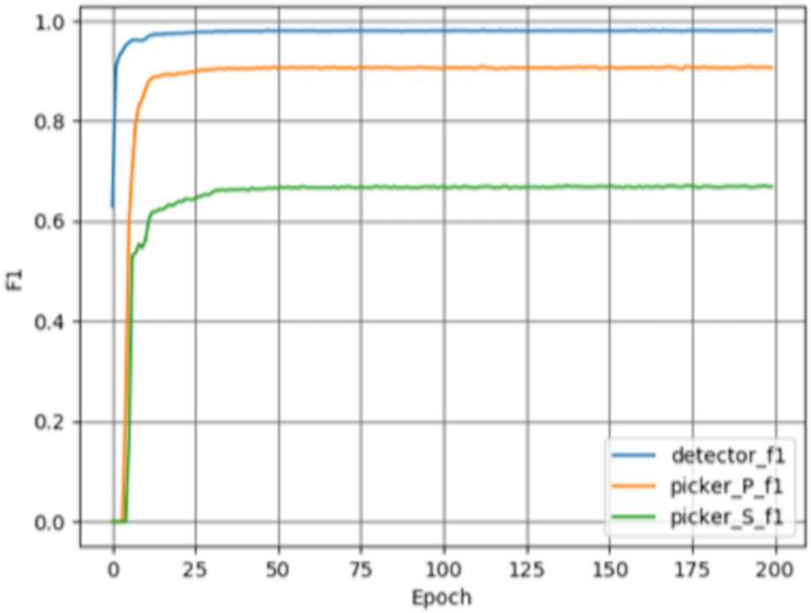

Supplementary Figure 2. Training learning curves. The training curves in terms of F1-score and as a function of epochs are provided for detection, P-wave picker, and S-wave picker decoders. Each of these decoders has its own loss function and their training process is independent from others decoder branches.

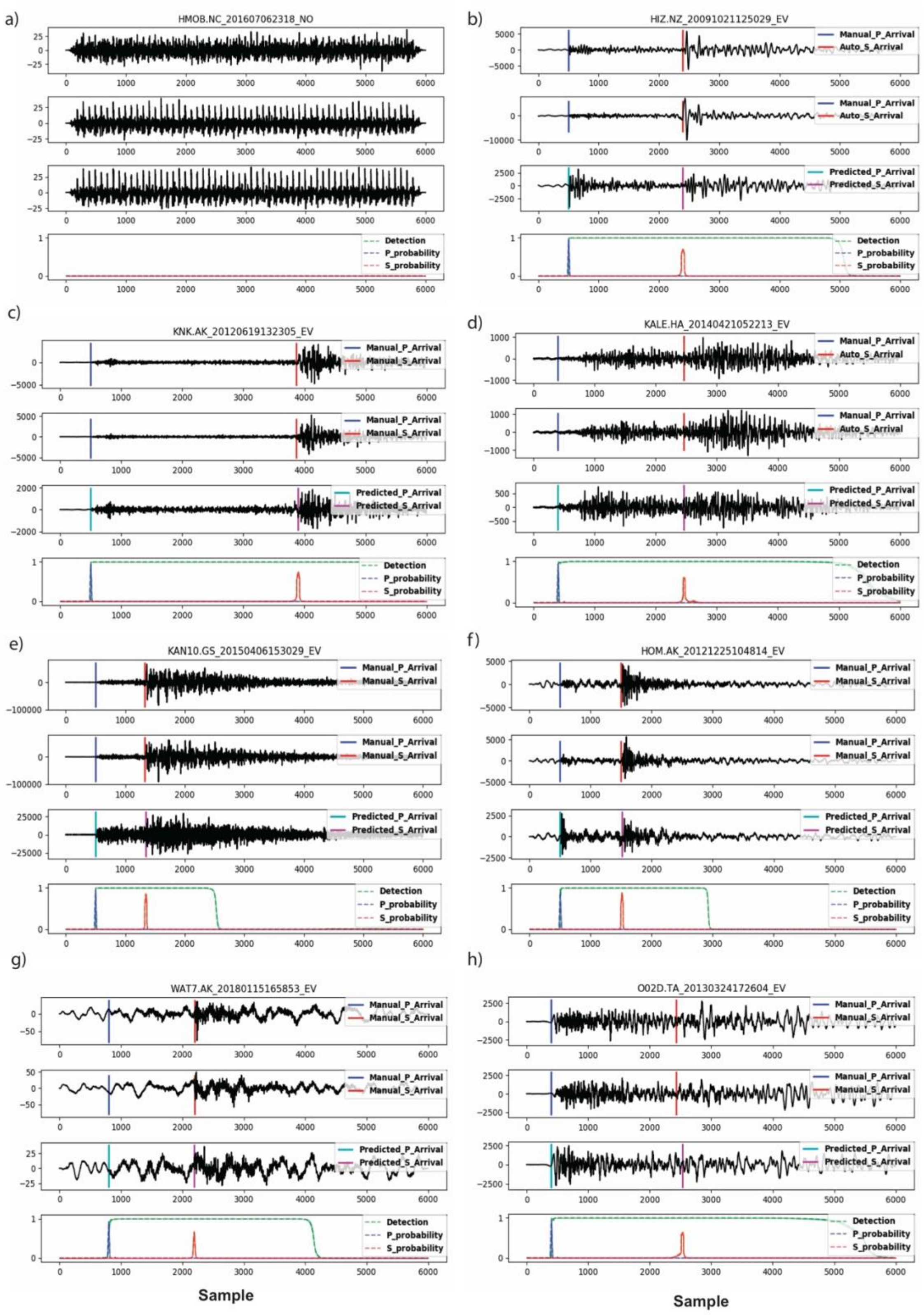

Supplementary Figure 3. Samples of test resultsv1. 8 representative waveform presenting performance of the model on different types of data in the test set. Each waveform is 60 seconds long with 100 sample per second.

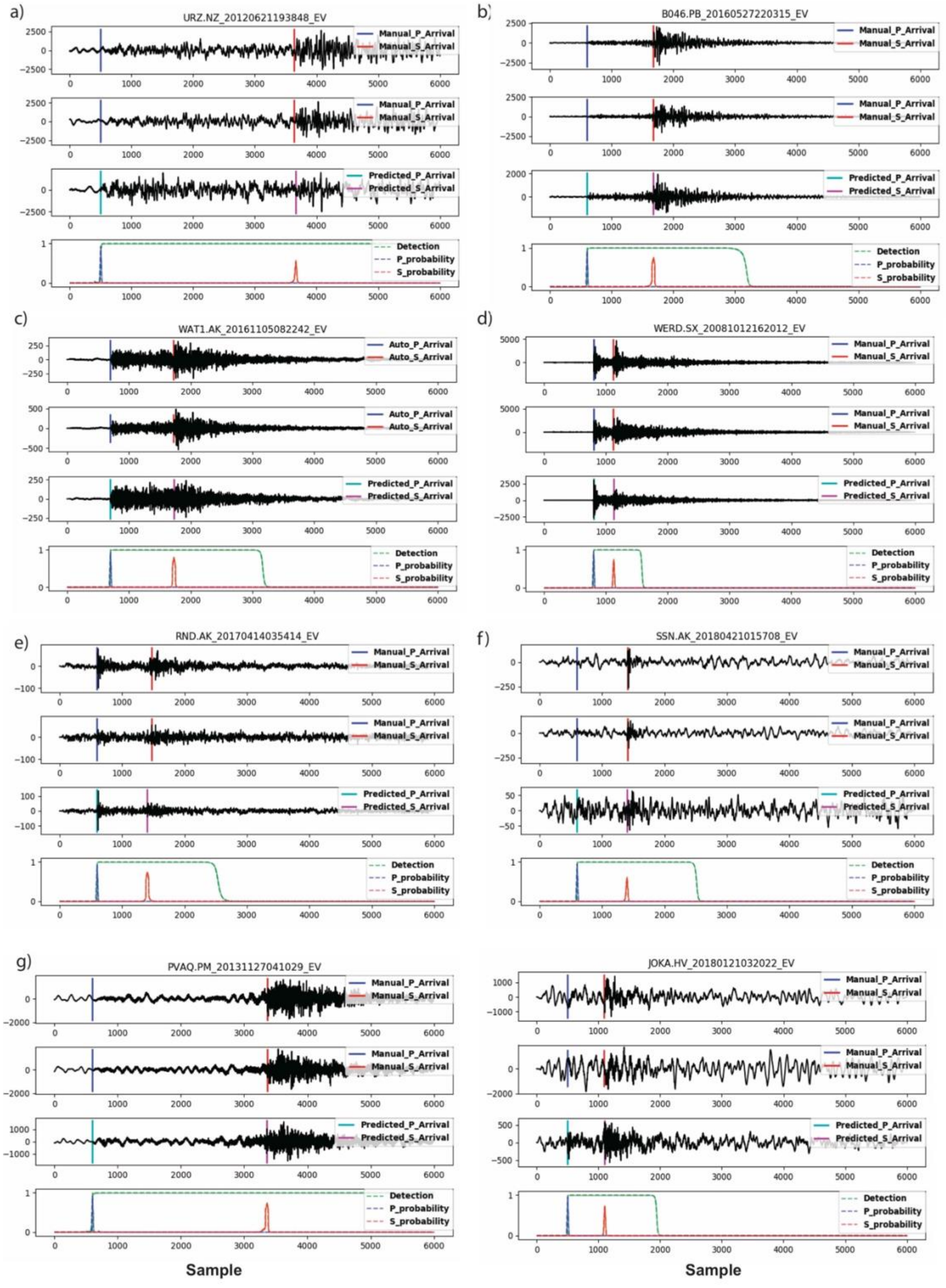

Supplementary Figure 4. Samples of test results 2. 8 representative waveform presenting performance of the model on different types of data in the test set. Each waveform is 60 seconds long with 100 sample per second.

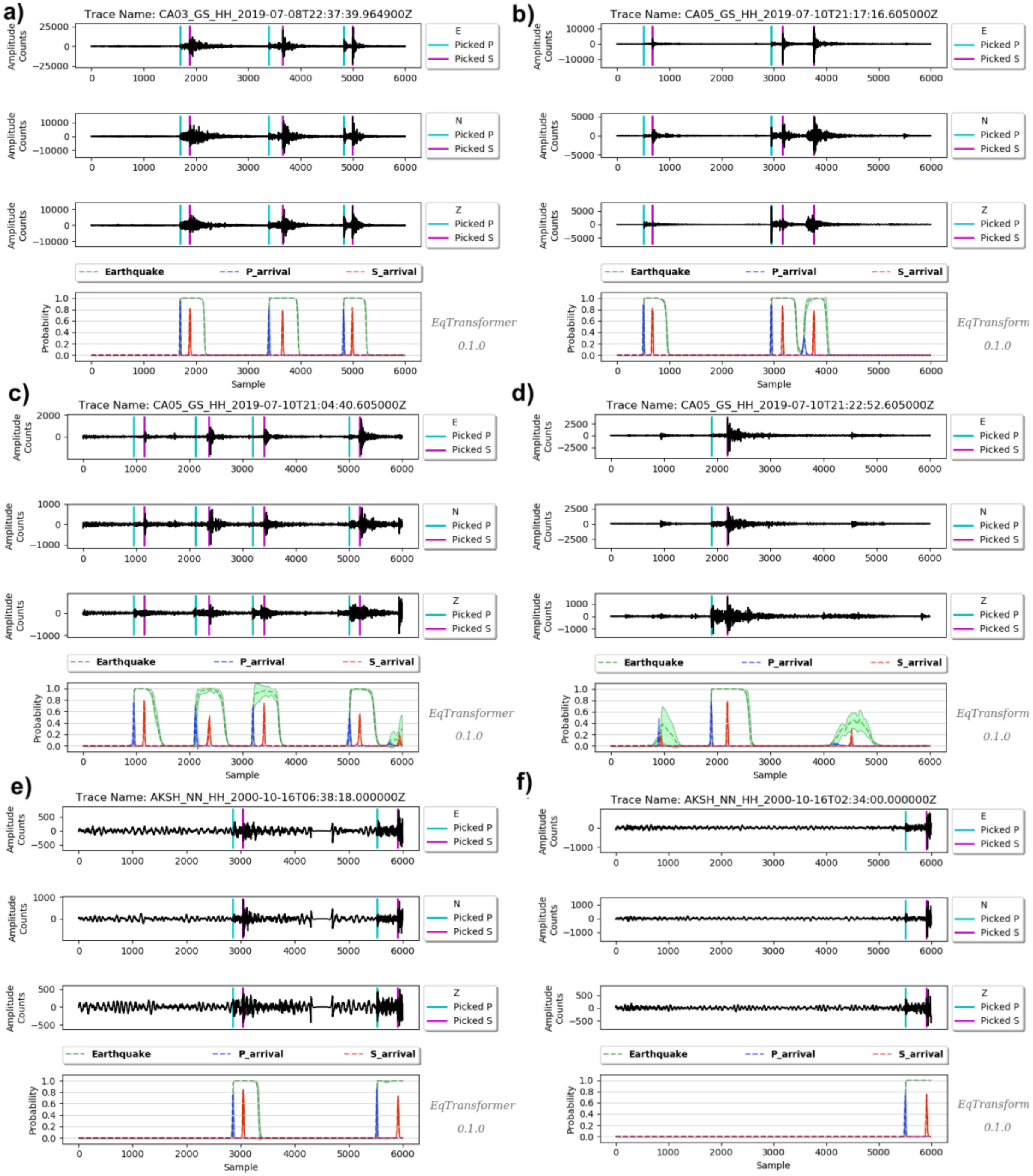

Supplementary Figure 5. Samples of test results 3. 6 representative waveform presenting performance of the model on continuous data recorded in Ridgecrest, California.

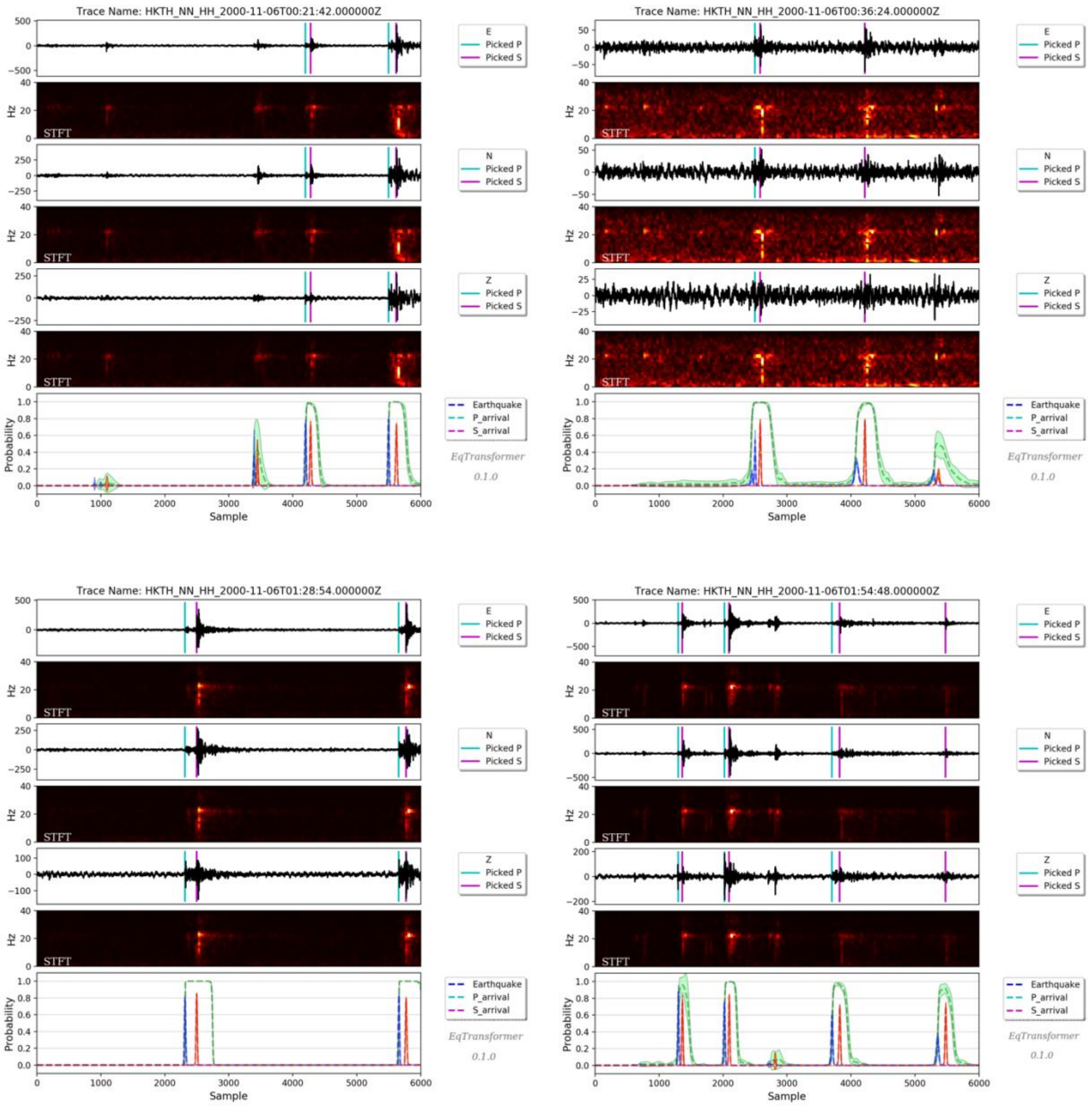

Supplementary Figure 6. Samples of test results 4. 4 representative waveform presenting performance of the model on continuous data recorded in Tottori, Japan.

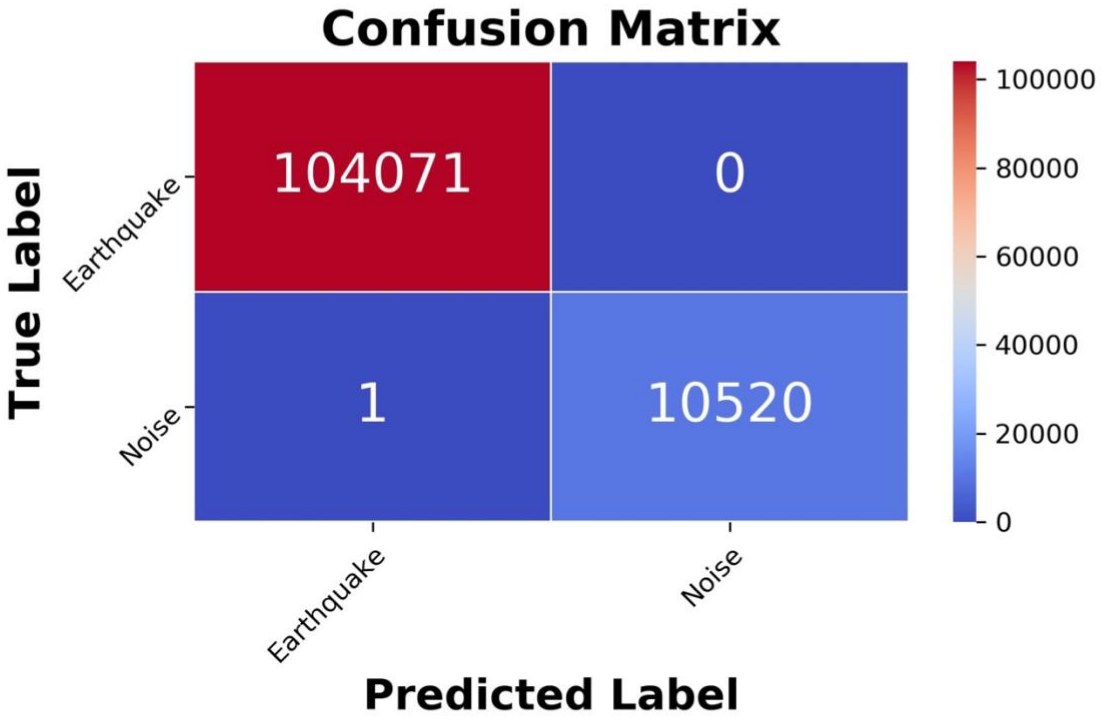

Supplementary Figure 7. The detection confusion matrix. Detection confusion matrix for the proposed method based on a threshold value of 0.5.

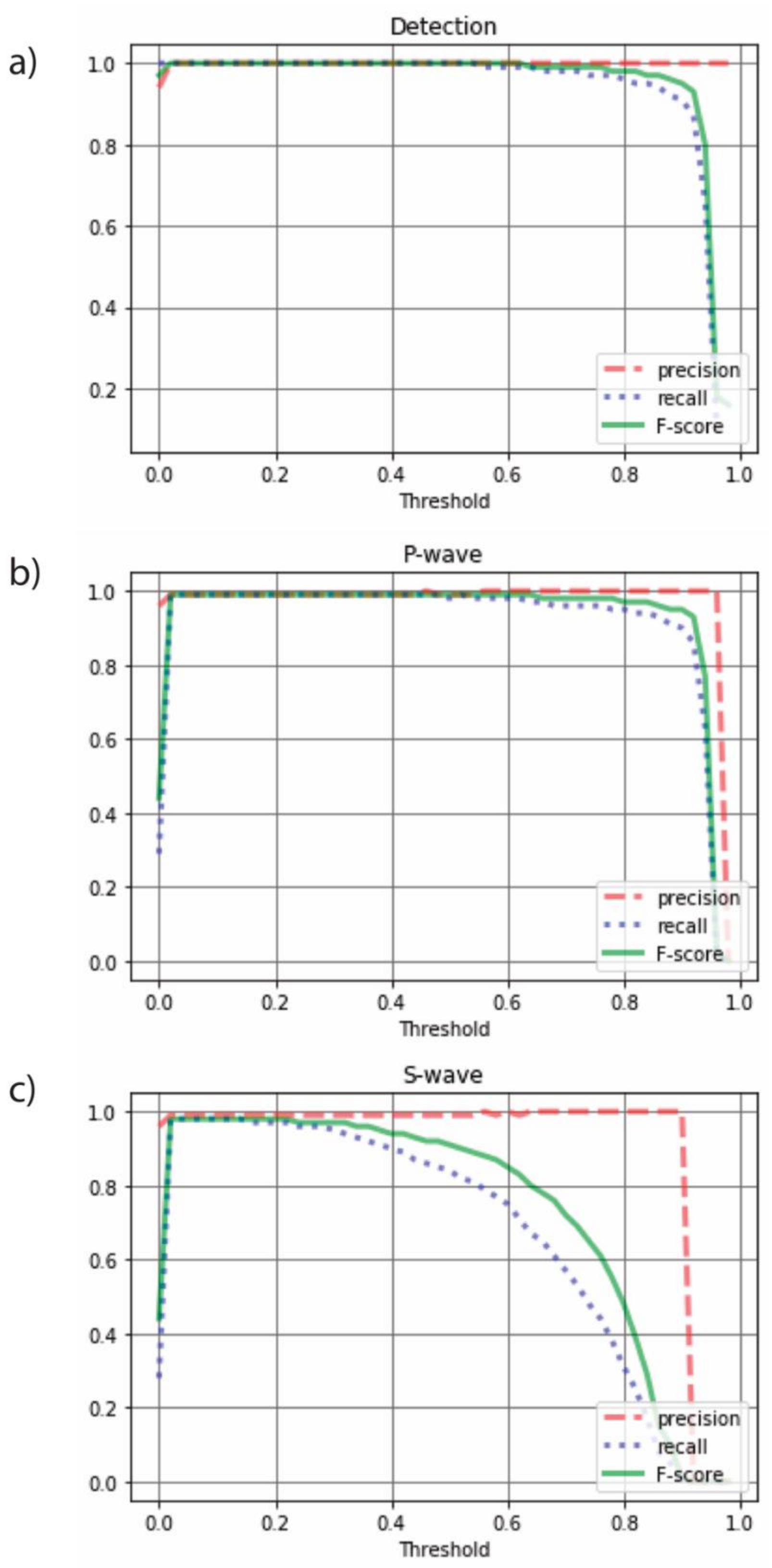

Supplementary Figure 8. The sensitivity test. Precision, Recall, and F1-score as a function of threshold value for detection (a), P-picking (b), and S-picking (c).

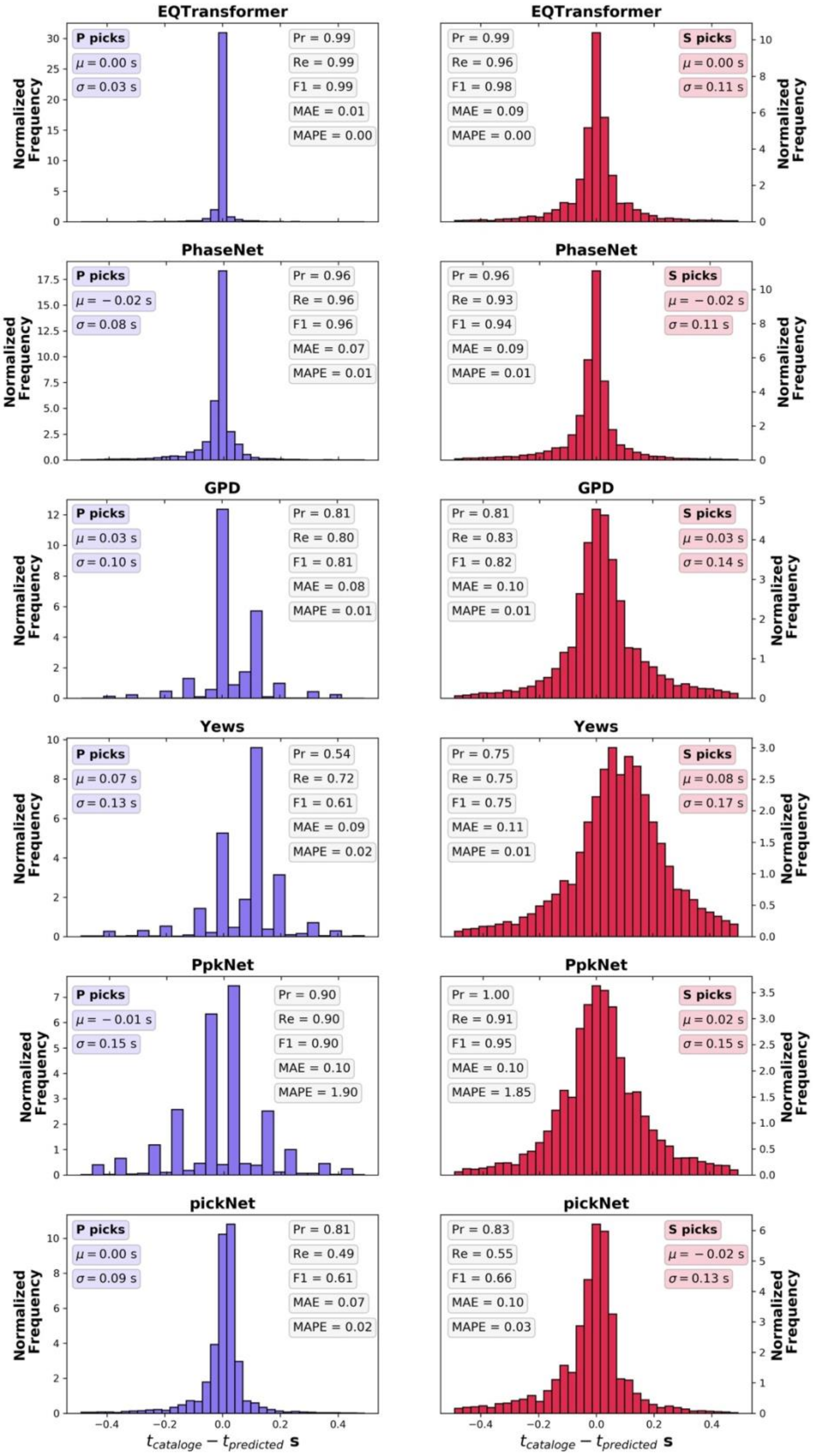
EQTransformer
P picks
μ = 0.00 s
σ = 0.03 s
Pr = 0.99
Re = 0.99
F1 = 0.99
MAE = 0.01
MAPE = 0.00
EQTransformer
Pr = 0.99
Re = 0.96
F1 = 0.98
MAE = 0.09
MAPE = 0.00
S picks
μ = 0.00 s
σ = 0.11 s
PhaseNet
P picks
μ = −0.02 s
σ = 0.08 s
Pr = 0.96
Re = 0.96
F1 = 0.96
MAE = 0.07
MAPE = 0.01
PhaseNet
Pr = 0.96
Re = 0.93
F1 = 0.94
MAE = 0.09
MAPE = 0.01
S picks
μ = −0.02 s
σ = 0.11 s
GPD
P picks
μ = 0.03 s
σ = 0.10 s
Pr = 0.81
Re = 0.80
F1 = 0.81
MAE = 0.08
MAPE = 0.01
GPD
Pr = 0.81
Re = 0.83
F1 = 0.82
MAE = 0.10
MAPE = 0.01
S picks
μ = 0.03 s
σ = 0.14 s
Yews
P picks
μ = 0.07 s
σ = 0.13 s
Pr = 0.54
Re = 0.72
F1 = 0.61
MAE = 0.09
MAPE = 0.02
Yews
Pr = 0.75
Re = 0.75
F1 = 0.75
MAE = 0.11
MAPE = 0.01
S picks
μ = 0.08 s
σ = 0.17 s
PpkNet
P picks
μ = −0.01 s
σ = 0.15 s
Pr = 0.90
Re = 0.90
F1 = 0.90
MAE = 0.10
MAPE = 1.90
PpkNet
Pr = 1.00
Re = 0.91
F1 = 0.95
MAE = 0.10
MAPE = 1.85
S picks
μ = 0.02 s
σ = 0.15 s
pickNet
P picks
μ = 0.00 s
σ = 0.09 s
Pr = 0.81
Re = 0.49
F1 = 0.61
MAE = 0.07
MAPE = 0.02
pickNet
Pr = 0.83
Re = 0.55
F1 = 0.66
MAE = 0.10
MAPE = 0.03
S picks
μ = −0.02 s
σ = 0.13 s
Normalized Frequency
t_cataloge − t_predicted s

Supplementary Figure 9. The comparison of distributions of picking errors with deep-learning methods. Comparison of picking performance (P waves in purple and S waves in red) of the proposed method (EQTransformer) with four different deep-learning-based pickers, PhaseNet, GPD, PpkNet, Yews , PickNet. Mean (μ) and standard deviation (σ) of each error distribution and the precision (Pr), recall (Re), F1-score (F1), mean average error (MAE), and the mean average percentage error (MAPE) are given in each subplot. A pick is considered as a true positive when its absolute distance from the ground truth is less than 0.5 second.

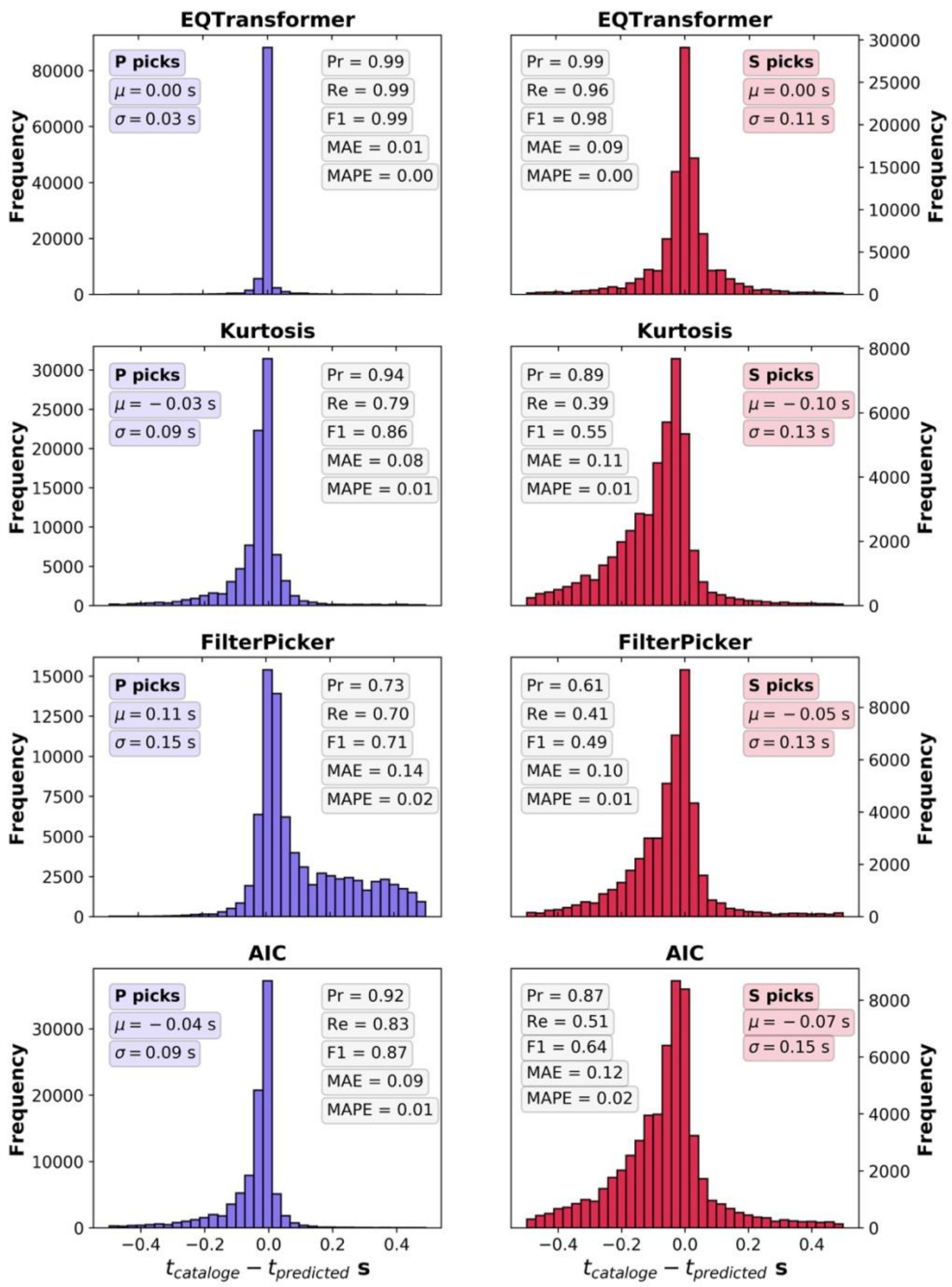

Supplementary Figure 10. The comparison of distributions of picking errors with traditional methods. Comparison of picking performance (P waves in purple and S waves in red) of the proposed method (EQTransformer) with three different traditional pickers, the Kurtosis, the FilterPicker, and Akaike Information Criteria (AIC). Mean (μ) and standard deviation (σ) of each error distribution and the precision (Pr), recall (Re), F1-score (F1), mean average error (MAE), and the mean average percentage error (MAPE) are given in each subplot. A pick is considered as a true positive when its absolute distance from the ground truth is less than 0.5 second.

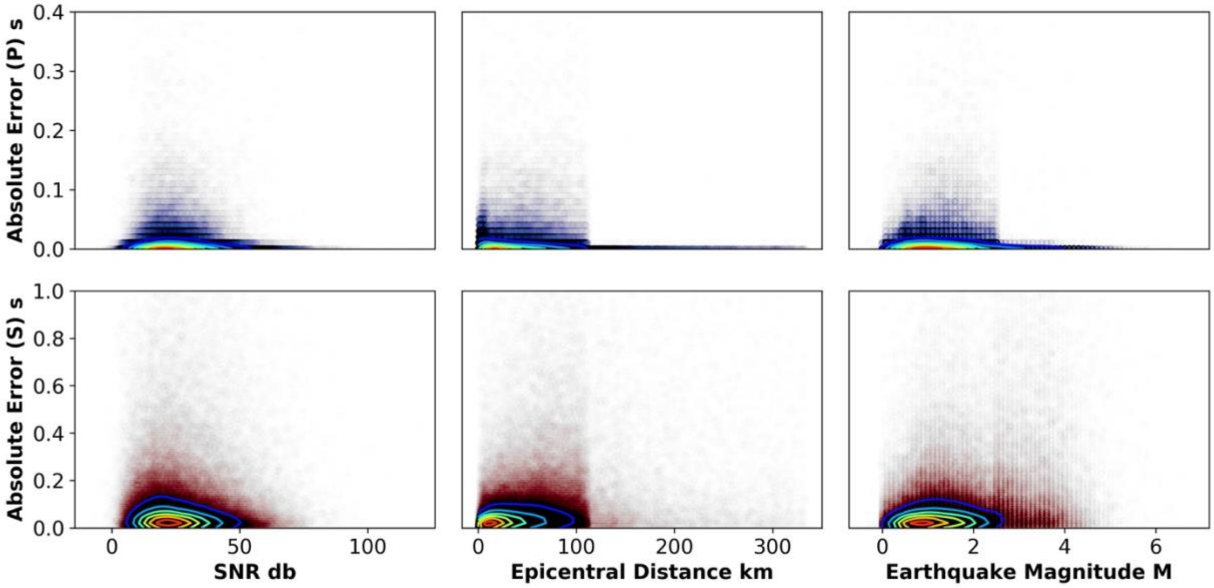

Supplementary Figure 11. Picking errors as functions of signal-to-noise ratio, distance, and magnitudes. Relations between errors for picking P and S phase and SNR, epicentral distance, and magnitude. Values for P are shown in blue and for S in red in the background while their density(or counts) are depicted with color coded contours

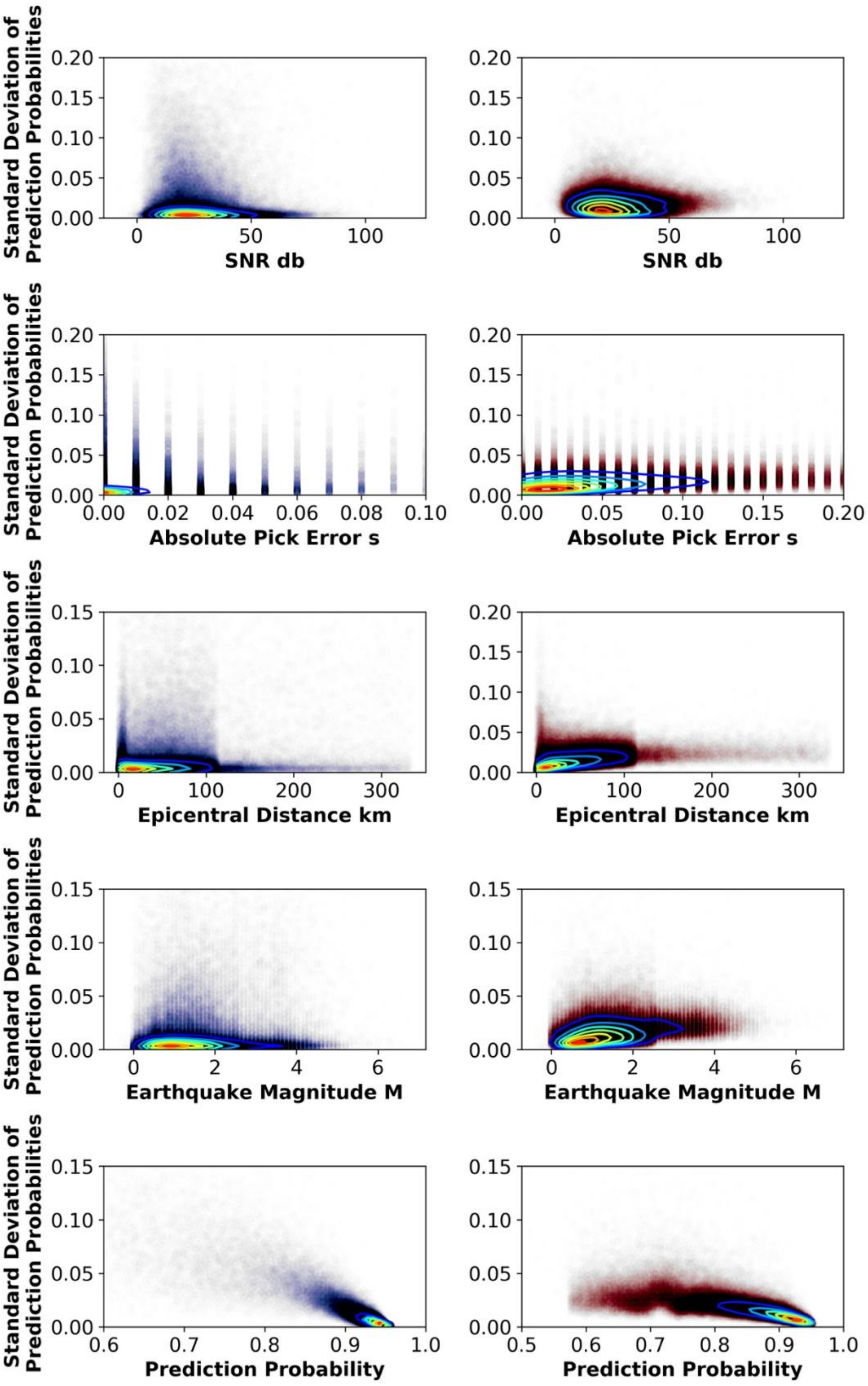
Standard Deviation of Prediction Probabilities
SNR db
Standard Deviation of Prediction Probabilities
SNR db
Standard Deviation of Prediction Probabilities
Absolute Pick Error s
Standard Deviation of Prediction Probabilities
Absolute Pick Error s
Standard Deviation of Prediction Probabilities
Epicentral Distance km
Standard Deviation of Prediction Probabilities
Epicentral Distance km
Standard Deviation of Prediction Probabilities
Earthquake Magnitude M
Standard Deviation of Prediction Probabilities
Earthquake Magnitude M
Standard Deviation of Prediction Probabilities
Prediction Probability
Standard Deviation of Prediction Probabilities
Prediction Probability

Supplementary Figure 12. Relations between estimated uncertainties for picking and characteristics of data and model. The estimated model uncertainties for P wave picks (in blue) and S wave picks (in red) as a function of: SNR, absolute error, epicentral distance, magnitude, and prediction probabilities. Values for P are shown in blue and for S in red in the background while their density(or counts) are depicted with color coded contours

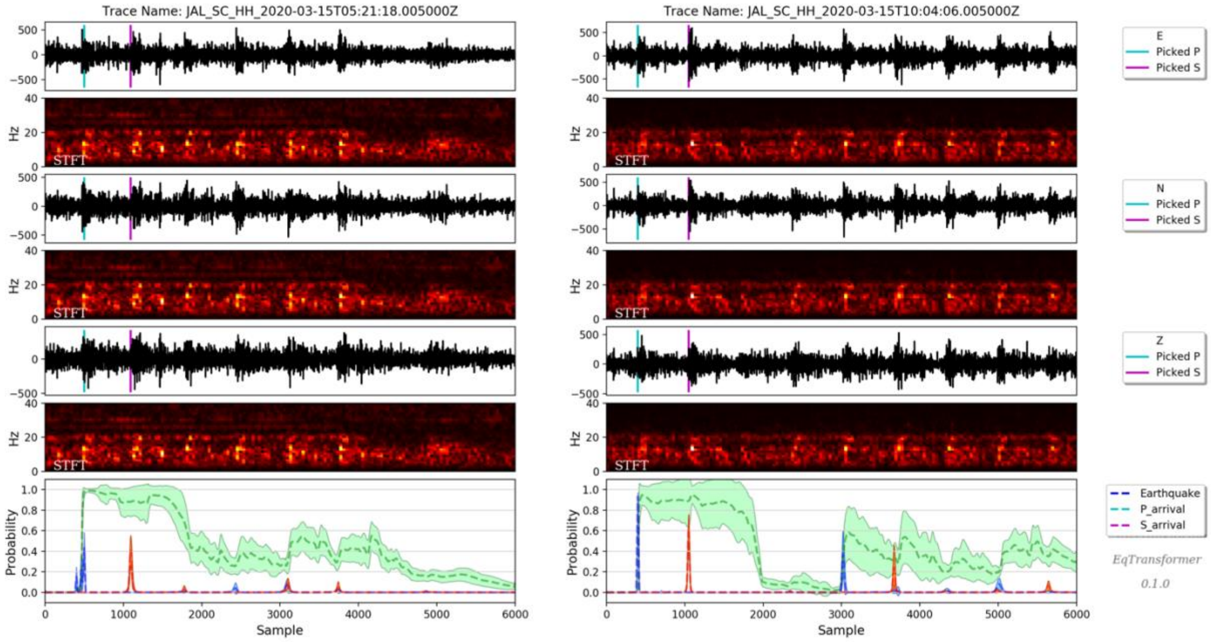

Supplementary Figure 13. Examples of false positives. Two examples of false positives due to periodic high-frequency cultural noise recorded by a station in Western Texas. Although the predicted probabilities for both detection and picking are relatively high, detection probabilities exhibit higher variations due to the model uncertainty.

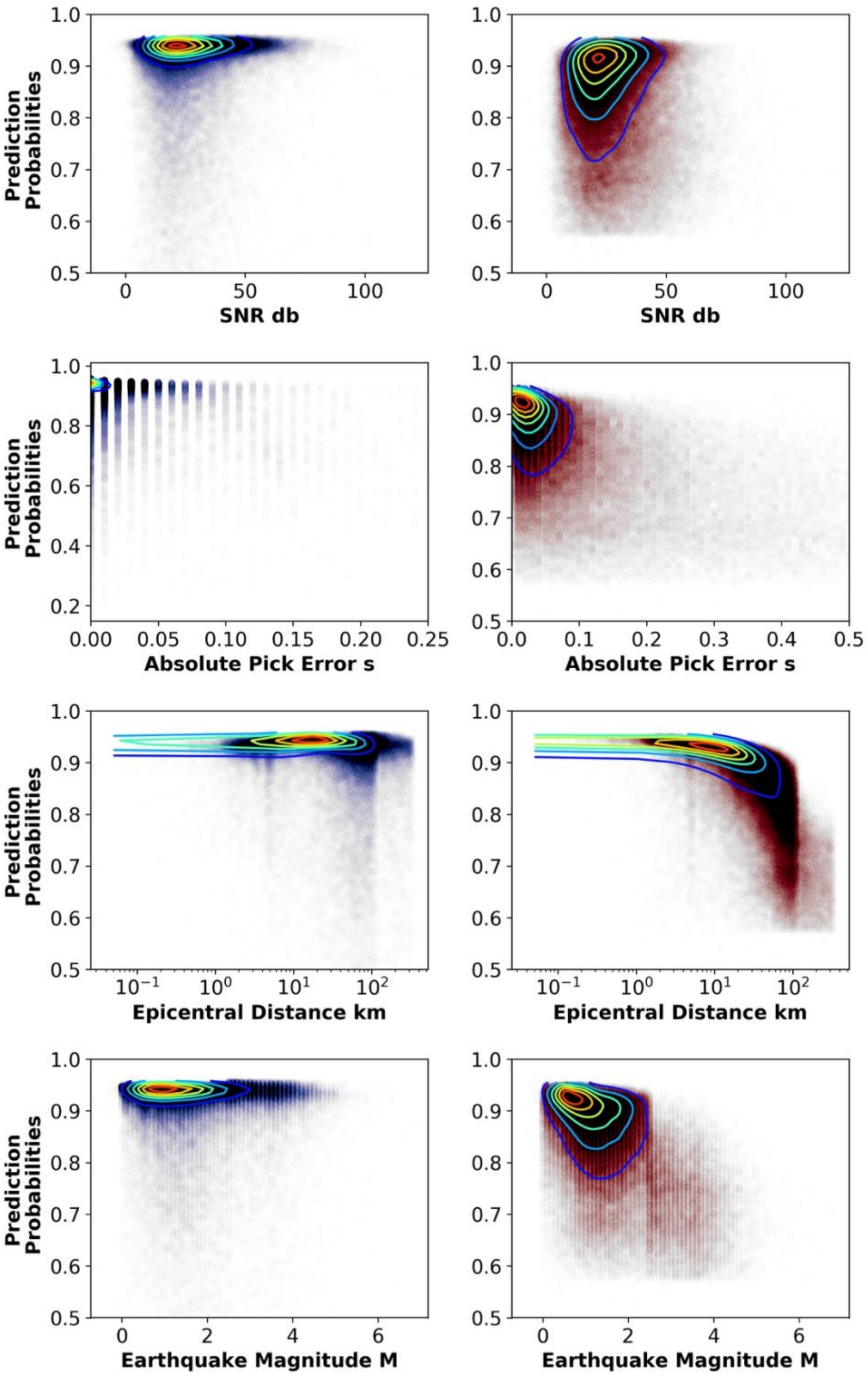

Supplementary Figure 14. Influences of different parameters on the output probabilities for picking. Relations between prediction probabilities for picking P and S phase and SNR, absolute error, epicentral distance, and magnitude. Values for P are shown in blue and for S in red in the background while their density(or counts) are depicted with color coded contours

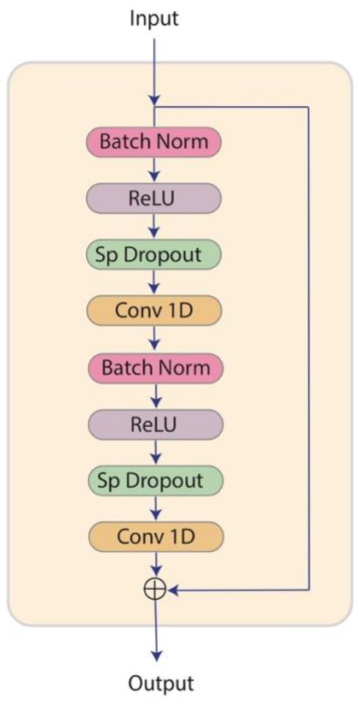

Supplementary Figure 15. Residual convolutional neural network (ResCNN) blocks. ResCNN blocks used in the encoder. Spatial dropout (Sp Dropout) layers have been used after each ReLU activation layer which is preceded by a batch normalization (Batch Norm).

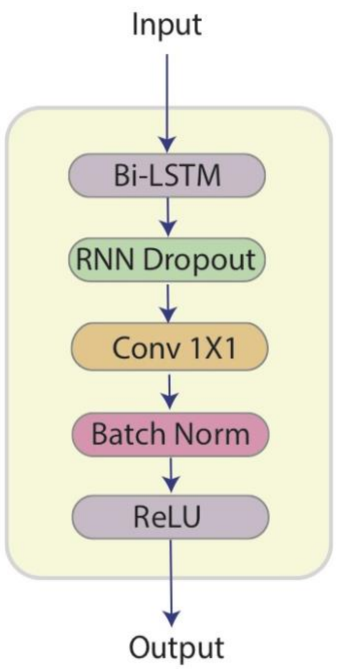

Supplementary Figure 16. Bilateral Long-Short-Term-Memory/Network in Network block (Bi-LSTM/NiN). Bi-LSTM/NiN blocks including a Bidirectional LSTM, one convolution layer

with one filter sized 1, batch normalization, ReLU activation layer. RNN Dropout is the recurrent dropout

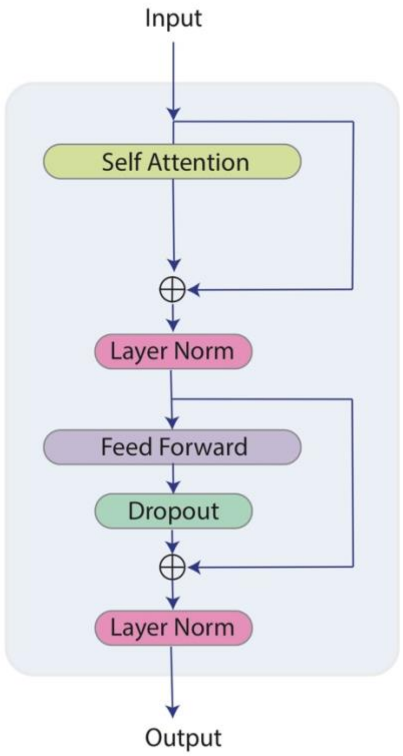

Supplementary Figure 17. Transformer. Single-head self-attention block for global attention. It includes one position-wise feed-forward network and layer normalization.
