# Supplementary material for: Earthquake transformer—an attentive deep-learning model for simultaneous earthquake detection and phase picking
Source: Nat Commun. 2020 Aug 7;11:3952. doi: 10.1038/s41467-020-17591-w (PMC7415159; doi:10.1038/s41467-020-17591-w)
Supplement: Supplementary file 3 — Description of Additional Supplementary Files [file 41467_2020_17591_MOESM3_ESM.pdf]

Descriptions of Additional Supplementary Files

Movie 01

Description: Evolution of the seismicity (detected and located in our study) in the Tottori area in map view

Movie 01

Description: Evolution of the seismicity (detected and located in our study) in the Tottori area in cross-section.
